# 3,3,4,4,5,5-Hexa­fluoro-1,2-bis[4-(phenyl­ethyn­yl)phen­yl]­cyclo­pentene

**DOI:** 10.1107/S1600536809052040

**Published:** 2009-12-12

**Authors:** Shaoqian Liu, Bing Chen

**Affiliations:** a Jiangxi Key Laboratory of Organic Chemistry, Jiangxi Science & Technology Normal University, Nanchang 330013, People’s Republic of China

## Abstract

The title compound, C_33_H_18_F_6_, has a V-shaped conjugated subunit. The dihedral angles between the central cyclo­pentene ring and the directly attached benzene rings are 32.7 (2) and 53.7 (2)°, respectively. The fluoro substituents are disordered, the occupancies refined to a 0.675 (2):0.325 (2) ratio.

## Related literature

The title compound was synthesised with the aim of simulating the characteristics of so-called left-handed materials on a mol­ecular scale. For a theoretical description of left-handed materials, see: Veselago (1968 ▶). For experimentally observed left-handed materials, see: Shelby *et al.* (2000 ▶); Chen *et al.* (2004 ▶); Zhou *et al.* (2006 ▶); Zhang *et al.* (2005 ▶); Liu *et al.* (2007 ▶).
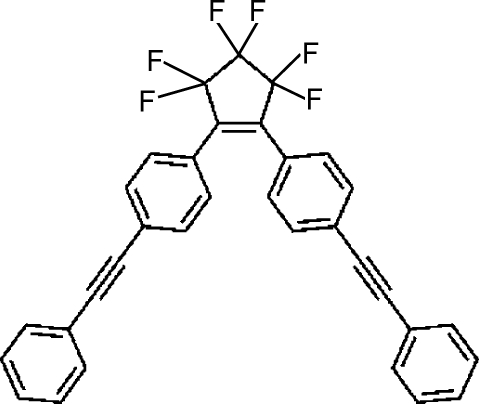


## Experimental

### 

#### Crystal data

C_33_H_18_F_6_
*M* *_r_* = 528.47
Monoclinic, 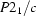

*a* = 16.350 (1) Å
*b* = 9.2893 (7) Å
*c* = 17.980 (1) Å
β = 103.654 (1)°
*V* = 2653.6 (4) Å^3^
*Z* = 4
Mo *K*α radiation
μ = 0.11 mm^−1^
*T* = 296 K
0.45 × 0.41 × 0.38 mm

#### Data collection

Bruker APEXII CCD diffractometer
Absorption correction: multi-scan (*SADABS*; Sheldrick, 1996 ▶) *T*_min_ = 0.954, *T*_max_ = 0.961
22979 measured reflections
6087 independent reflections
3767 reflections with *I* > 2σ(*I*)
*R*_int_ = 0.026

#### Refinement

*R*[*F* ^2^ > 2σ(*F* ^2^)] = 0.042
*wR*(*F* ^2^) = 0.109
*S* = 1.01
6087 reflections
407 parameters
H-atom parameters constrained
Δρ_max_ = 0.14 e Å^−3^
Δρ_min_ = −0.19 e Å^−3^

### 

Data collection: *APEX2* (Bruker, 2004 ▶); cell refinement: *SAINT* (Bruker, 2004 ▶); data reduction: *SAINT*; program(s) used to solve structure: *SHELXS97* (Sheldrick, 2008 ▶); program(s) used to refine structure: *SHELXL97* (Sheldrick, 2008 ▶); molecular graphics: *SHELXTL* (Sheldrick, 2008 ▶); software used to prepare material for publication: *SHELXTL*.

## Supplementary Material

Crystal structure: contains datablocks I, global. DOI: 10.1107/S1600536809052040/im2157sup1.cif

Structure factors: contains datablocks I. DOI: 10.1107/S1600536809052040/im2157Isup2.hkl

Additional supplementary materials:  crystallographic information; 3D view; checkCIF report

## Figures and Tables

**Fig. 1.**
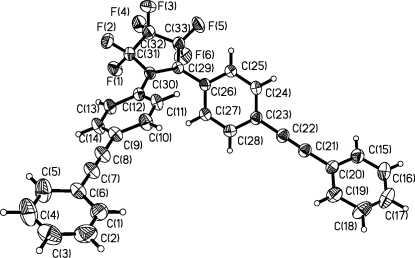
Molecular structure showing the atom-labelling scheme. Ellipsoids are drawn at the 30% probability level. H atoms are represented as small spheres of arbitrary radii.

## supplementary crystallographic information

### Comment

Left-handed material is a kind of material that exhibits a negative refraction index. It has been explored theoretically by Veselago (1968). In 2000, a two-dimensional isotropic left-handed material was verified showing a negative refraction index (Shelby *et al.*, 2000). From then on, the study of left-handed materials has gained increasing interest in physics-related fields. So far, most of the left-handed materials were realised *via* micro-processing technologies to integrate a metal into a S-shape (Chen *et al.*, 2004) or H-shape (Zhou *et al.*, 2006) micro-structural unit, therefore achieving a periodic arrangement. However, due to the technology limitations of nano-scale processing technology, most of the successful experimental verification are carried out in the microwave band (Zhang *et al.*, 2005; Liu *et al.*, 2007). Only if the left-handed material structural unit achieves a smaller size, a negative refraction index might be realised in the visible range. Using concepts of chemical synthesis technology to create structural features of left-handed materials at the molecular level might show potential to realise negative refraction in the visible range. The molecular model should exhibit an extended conjugated system providing electrons that are easily excited.

In this article, we tried to simulate structural features of left-handed material and designed a V-shaped conjugated organic molecular structure (Fig. 1). The title compound turns out to be a single-crystal, but whether it is really close to the structural features of left-handed materials or not and how to modify it to exhibit negative refraction, is still under investigation. The dihedral angle between the benzene ring C9—C14 and the adjacent benzene ring C1—C6 is 31.4 (1) °, the dihedral angle between the benzene ring C23—C28 and the adjacent benzene ring C15—C20 is 50.7 (2) °. C7—C8 (1.192 (2) Å) and C21—C22 (1.188 (2) Å) are acetylenic bonds and are therefore significantly shorter than the other carbon carbon bonds. The fluoro substituents at the cyclopentene ring are disordered with occupancies refined to a 0.675 (2):0.325 (2) ratio.

### Experimental

Synthesis of the title compound was carried out in two steps:(1) 4-bromo-1-phenylethynylbenzene,was obtained in 72.6% yield by the reaction of phenylacetylene (12 g, 0.1 mol) and 1-Bromo-4-iodobenzene (28.29, 0.1 mol) in N(C_2_H_5_)_3_ (150 ml) solution catalyzed by CuI (0.3 g, 1.55 mmol), PPh_3_ (0.6 g, 2.45 mmol) and Pd(PPh_3_)Cl_2_(0.3 g, 0.42 mmol) at 336 K; (2) 1.6 ml of n-BuLi/hexane solution (2.5 mol/*L*, 4.0 mmol) was added slowly at 195 K under a nitrogen atmosphere to a stirred THF solution (40 ml) containing 4-bromo-1-phenylethynylbenzene (1.03 g, 4 mmol). After 30 min perfluorocyclopentene (0.27 ml, 2 mmol) was added and the mixture was stirred for 1 h at this temperature. The reaction mixture was extracted with CHCl_3_, evaporated *in vacuo* and purifed by column chromatography (hexane) to give the title compound. After recrystallization from CH_2_Cl_2_ light yellow-green crystals of the title compound were obtained in 23.4% yield. ^1^HNMR (400 MHz, CDCl_3_): δ 7.35(m, 10H), 7.51 (m, 8H)

### Refinement

All H atoms attached to C were fixed geometrically and treated as riding with C—H = 0.93 Å and *U*_iso_(H) = 1.2*U*_eq_(C). The occupancies of the disordered components refined to a 0.675 (2):0.325 (2) ratio for F1:F1', F2:F2', F3:F3', F4:F4' F5:F5' and F6:F6'.

### Crystal data

**Table:**

C_33_H_18_F_6_	*F*(000) = 1080
*M**_r_* = 528.47	*D*_x_ = 1.323 Mg m^−^^3^
Monoclinic, *P*2_1_/*c*	Mo *K*α radiation, λ = 0.71073 Å
Hall symbol: -P 2ybc	Cell parameters from 6530 reflections
*a* = 16.350 (1) Å	θ = 2.3–25.8°
*b* = 9.2893 (7) Å	µ = 0.11 mm^−^^1^
*c* = 17.980 (1) Å	*T* = 296 K
β = 103.654 (1)°	Block, yellow
*V* = 2653.6 (4) Å^3^	0.45 × 0.41 × 0.38 mm
*Z* = 4	

### Data collection

**Table:**

Bruker APEXII CCD diffractometer	6087 independent reflections
Radiation source: fine-focus sealed tube	3767 reflections with *I* > 2σ(*I*)
graphite	*R*_int_ = 0.026
φ and ω scans	θ_max_ = 27.5°, θ_min_ = 2.3°
Absorption correction: multi-scan (*SADABS*; Sheldrick, 1996)	*h* = −21→21
*T*_min_ = 0.954, *T*_max_ = 0.961	*k* = −12→12
22979 measured reflections	*l* = −23→23

### Refinement

**Table:**

Refinement on *F*^2^	Primary atom site location: structure-invariant direct methods
Least-squares matrix: full	Secondary atom site location: difference Fourier map
*R*[*F*^2^ > 2σ(*F*^2^)] = 0.042	Hydrogen site location: inferred from neighbouring sites
*wR*(*F*^2^) = 0.109	H-atom parameters constrained
*S* = 1.01	*w* = 1/[σ^2^(*F*_o_^2^) + (0.0298*P*)^2^ + 0.8178*P*] where *P* = (*F*_o_^2^ + 2*F*_c_^2^)/3
6087 reflections	(Δ/σ)_max_ = 0.002
407 parameters	Δρ_max_ = 0.14 e Å^−^^3^
0 restraints	Δρ_min_ = −0.19 e Å^−^^3^

### Special details

**Table:**

Geometry. All e.s.d.'s (except the e.s.d. in the dihedral angle between two l.s. planes) are estimated using the full covariance matrix. The cell e.s.d.'s are taken into account individually in the estimation of e.s.d.'s in distances, angles and torsion angles; correlations between e.s.d.'s in cell parameters are only used when they are defined by crystal symmetry. An approximate (isotropic) treatment of cell e.s.d.'s is used for estimating e.s.d.'s involving l.s. planes.
Refinement. Refinement of *F*^2^ against ALL reflections. The weighted *R*-factor *wR* and goodness of fit *S* are based on *F*^2^, conventional *R*-factors *R* are based on *F*, with *F* set to zero for negative *F*^2^. The threshold expression of *F*^2^ > σ(*F*^2^) is used only for calculating *R*-factors(gt) *etc*. and is not relevant to the choice of reflections for refinement. *R*-factors based on *F*^2^ are statistically about twice as large as those based on *F*, and *R*- factors based on ALL data will be even larger.

### Fractional atomic coordinates and isotropic or equivalent isotropic displacement parameters (Å^2^)

**Table:**

	*x*	*y*	*z*	*U*_iso_*/*U*_eq_	Occ. (<1)
C26	0.19961 (9)	1.02109 (17)	1.03064 (9)	0.0496 (4)	
C22	0.10730 (11)	0.6218 (2)	0.93186 (10)	0.0611 (4)	
C23	0.13842 (10)	0.75808 (18)	0.96494 (9)	0.0557 (4)	
C12	0.36369 (9)	1.19273 (17)	1.01487 (9)	0.0512 (4)	
C7	0.55877 (11)	1.1281 (2)	0.81141 (10)	0.0687 (5)	
C20	0.04809 (11)	0.37578 (18)	0.87084 (9)	0.0557 (4)	
C11	0.33280 (10)	1.1354 (2)	0.94246 (9)	0.0628 (5)	
H11	0.2761	1.1117	0.9268	0.075*	
C9	0.46915 (10)	1.1503 (2)	0.91437 (10)	0.0623 (5)	
C21	0.08196 (11)	0.5089 (2)	0.90495 (10)	0.0596 (4)	
C27	0.24980 (10)	0.89899 (19)	1.04209 (10)	0.0587 (4)	
H27	0.3046	0.9051	1.0717	0.070*	
C28	0.21994 (11)	0.76934 (19)	1.01039 (10)	0.0618 (4)	
H28	0.2544	0.6884	1.0193	0.074*	
C8	0.52015 (11)	1.1357 (2)	0.85985 (10)	0.0690 (5)	
C24	0.08860 (10)	0.88041 (19)	0.95314 (10)	0.0625 (5)	
H24	0.0342	0.8748	0.9226	0.075*	
C25	0.11797 (10)	1.01009 (19)	0.98572 (10)	0.0594 (4)	
H25	0.0832	1.0906	0.9777	0.071*	
C6	0.60588 (10)	1.1192 (2)	0.75391 (10)	0.0646 (5)	
C14	0.50063 (11)	1.2050 (2)	0.98687 (11)	0.0707 (5)	
H14	0.5575	1.2280	1.0024	0.085*	
C13	0.44917 (10)	1.2260 (2)	1.03648 (10)	0.0647 (5)	
H13	0.4718	1.2630	1.0851	0.078*	
C15	−0.02972 (12)	0.3286 (2)	0.87935 (11)	0.0707 (5)	
H15	−0.0586	0.3821	0.9086	0.085*	
C19	0.09035 (14)	0.2938 (2)	0.82795 (11)	0.0771 (6)	
H19	0.1431	0.3225	0.8226	0.093*	
C10	0.38442 (11)	1.1131 (2)	0.89358 (10)	0.0689 (5)	
H10	0.3624	1.0725	0.8458	0.083*	
C1	0.58778 (14)	1.0158 (2)	0.69804 (13)	0.0854 (6)	
H1	0.5440	0.9514	0.6972	0.102*	
C16	−0.06433 (14)	0.2039 (3)	0.84499 (13)	0.0880 (6)	
H16	−0.1163	0.1727	0.8513	0.106*	
C17	−0.02255 (19)	0.1254 (3)	0.80152 (13)	0.0992 (8)	
H17	−0.0466	0.0417	0.7776	0.119*	
C4	0.71624 (17)	1.2032 (4)	0.69872 (17)	0.1292 (11)	
H4	0.7600	1.2673	0.6989	0.155*	
C18	0.05436 (19)	0.1691 (2)	0.79302 (13)	0.1001 (8)	
H18	0.0827	0.1147	0.7636	0.120*	
C2	0.63364 (18)	1.0065 (3)	0.64331 (15)	0.1086 (8)	
H2	0.6204	0.9364	0.6054	0.130*	
C5	0.67010 (15)	1.2145 (3)	0.75389 (14)	0.1059 (8)	
H5	0.6825	1.2864	0.7908	0.127*	
C3	0.69781 (18)	1.0985 (4)	0.64418 (16)	0.1128 (9)	
H3	0.7294	1.0904	0.6076	0.135*	
C29	0.23397 (9)	1.15683 (17)	1.06773 (9)	0.0498 (4)	
C30	0.30656 (9)	1.22316 (17)	1.06528 (9)	0.0497 (4)	
C31	0.32336 (11)	1.34507 (19)	1.12133 (10)	0.0607 (4)	
C33	0.19144 (12)	1.2310 (2)	1.12173 (11)	0.0687 (5)	
C32	0.24253 (12)	1.3637 (3)	1.14835 (12)	0.0749 (6)	
F1	0.3864 (5)	1.3132 (8)	1.1850 (5)	0.0784 (12)	0.675 (12)
F2	0.3498 (3)	1.4682 (7)	1.0946 (4)	0.0804 (11)	0.675 (12)
F5	0.1123 (2)	1.2516 (6)	1.1010 (4)	0.0953 (16)	0.675 (12)
F3	0.1990 (2)	1.4774 (4)	1.1027 (3)	0.0916 (12)	0.675 (12)
F4	0.2516 (2)	1.4078 (6)	1.2183 (3)	0.0956 (15)	0.675 (12)
F6	0.2036 (5)	1.1456 (4)	1.1900 (3)	0.0992 (13)	0.675 (12)
F3'	0.2714 (6)	1.301 (2)	1.2310 (3)	0.139 (5)	0.325 (12)
F4'	0.2128 (5)	1.4715 (12)	1.1595 (14)	0.148 (7)	0.325 (12)
F2'	0.3905 (11)	1.328 (2)	1.1726 (11)	0.120 (6)	0.325 (12)
F1'	0.3278 (12)	1.4677 (15)	1.0822 (11)	0.136 (5)	0.325 (12)
F5'	0.1528 (8)	1.1542 (8)	1.1592 (6)	0.092 (3)	0.325 (12)
F6'	0.1151 (6)	1.3026 (12)	1.0670 (6)	0.093 (3)	0.325 (12)

### Atomic displacement parameters (Å^2^)

**Table:**

	*U*^11^	*U*^22^	*U*^33^	*U*^12^	*U*^13^	*U*^23^
C26	0.0444 (8)	0.0530 (9)	0.0529 (9)	−0.0063 (7)	0.0145 (7)	0.0000 (7)
C22	0.0645 (10)	0.0591 (11)	0.0634 (11)	−0.0080 (9)	0.0224 (9)	−0.0028 (9)
C23	0.0567 (9)	0.0552 (10)	0.0585 (10)	−0.0098 (8)	0.0204 (8)	−0.0026 (8)
C12	0.0444 (8)	0.0555 (10)	0.0520 (9)	−0.0046 (7)	0.0082 (7)	0.0031 (8)
C7	0.0511 (9)	0.0925 (14)	0.0618 (11)	0.0041 (9)	0.0118 (9)	0.0063 (10)
C20	0.0668 (10)	0.0501 (9)	0.0509 (9)	−0.0051 (8)	0.0153 (8)	0.0017 (8)
C11	0.0453 (8)	0.0886 (13)	0.0534 (10)	−0.0120 (9)	0.0092 (7)	−0.0005 (9)
C9	0.0498 (9)	0.0783 (12)	0.0593 (10)	0.0031 (9)	0.0143 (8)	0.0063 (9)
C21	0.0644 (10)	0.0567 (11)	0.0602 (10)	−0.0057 (9)	0.0195 (8)	−0.0014 (9)
C27	0.0468 (9)	0.0619 (11)	0.0641 (10)	−0.0006 (8)	0.0066 (8)	−0.0017 (9)
C28	0.0605 (10)	0.0540 (10)	0.0703 (11)	0.0046 (8)	0.0142 (9)	0.0003 (9)
C8	0.0511 (9)	0.0932 (14)	0.0623 (11)	0.0046 (9)	0.0124 (9)	0.0069 (10)
C24	0.0453 (9)	0.0628 (11)	0.0758 (12)	−0.0087 (8)	0.0072 (8)	−0.0064 (9)
C25	0.0458 (9)	0.0555 (10)	0.0746 (11)	−0.0003 (8)	0.0094 (8)	−0.0026 (9)
C6	0.0513 (9)	0.0838 (13)	0.0604 (10)	0.0053 (9)	0.0163 (8)	0.0092 (10)
C14	0.0422 (8)	0.0988 (15)	0.0702 (12)	−0.0067 (9)	0.0113 (8)	−0.0038 (11)
C13	0.0472 (9)	0.0844 (13)	0.0604 (10)	−0.0097 (9)	0.0083 (8)	−0.0096 (9)
C15	0.0685 (11)	0.0666 (12)	0.0791 (13)	−0.0076 (10)	0.0218 (10)	−0.0046 (10)
C19	0.0946 (14)	0.0682 (13)	0.0806 (13)	−0.0088 (11)	0.0448 (12)	−0.0060 (11)
C10	0.0575 (10)	0.0963 (15)	0.0522 (10)	−0.0098 (10)	0.0117 (8)	−0.0062 (10)
C1	0.0902 (15)	0.0793 (14)	0.0971 (16)	−0.0048 (12)	0.0428 (13)	−0.0035 (13)
C16	0.0857 (14)	0.0859 (16)	0.0893 (15)	−0.0297 (12)	0.0142 (12)	−0.0059 (13)
C17	0.148 (2)	0.0765 (15)	0.0707 (14)	−0.0385 (16)	0.0214 (15)	−0.0146 (12)
C4	0.105 (2)	0.179 (3)	0.117 (2)	−0.048 (2)	0.0534 (18)	0.014 (2)
C18	0.159 (2)	0.0739 (15)	0.0832 (15)	−0.0110 (15)	0.0608 (16)	−0.0200 (12)
C2	0.138 (2)	0.0981 (18)	0.1097 (19)	0.0115 (17)	0.0701 (18)	−0.0086 (15)
C5	0.1026 (17)	0.132 (2)	0.0909 (16)	−0.0434 (16)	0.0391 (14)	−0.0131 (15)
C3	0.106 (2)	0.149 (3)	0.103 (2)	0.0215 (19)	0.0633 (17)	0.0258 (19)
C29	0.0447 (8)	0.0531 (9)	0.0512 (9)	−0.0019 (7)	0.0103 (7)	0.0012 (7)
C30	0.0443 (8)	0.0532 (9)	0.0486 (9)	−0.0021 (7)	0.0048 (7)	0.0020 (7)
C31	0.0520 (10)	0.0613 (11)	0.0656 (11)	−0.0086 (9)	0.0076 (9)	−0.0058 (9)
C33	0.0594 (11)	0.0736 (13)	0.0803 (13)	−0.0109 (10)	0.0304 (10)	−0.0131 (11)
C32	0.0639 (11)	0.0832 (15)	0.0783 (14)	−0.0063 (11)	0.0180 (10)	−0.0299 (12)
F1	0.059 (2)	0.105 (3)	0.0622 (17)	0.0063 (17)	−0.0038 (14)	−0.0136 (18)
F2	0.0774 (16)	0.063 (2)	0.107 (2)	−0.0201 (11)	0.0328 (14)	−0.0124 (16)
F5	0.0460 (12)	0.122 (3)	0.121 (4)	−0.0099 (17)	0.0250 (19)	−0.049 (3)
F3	0.0723 (13)	0.0717 (16)	0.131 (3)	0.0201 (11)	0.0252 (17)	0.0108 (17)
F4	0.0840 (19)	0.127 (4)	0.0782 (18)	−0.015 (2)	0.0248 (13)	−0.045 (2)
F6	0.133 (4)	0.1022 (16)	0.078 (2)	−0.008 (2)	0.057 (2)	0.0078 (15)
F3'	0.113 (4)	0.232 (15)	0.069 (3)	−0.042 (7)	0.016 (3)	−0.021 (5)
F4'	0.097 (5)	0.093 (6)	0.262 (19)	−0.011 (4)	0.059 (8)	−0.091 (9)
F2'	0.068 (5)	0.180 (13)	0.098 (9)	−0.020 (6)	−0.007 (5)	−0.075 (9)
F1'	0.204 (13)	0.046 (4)	0.164 (9)	−0.014 (6)	0.056 (9)	0.008 (5)
F5'	0.115 (6)	0.092 (3)	0.088 (5)	−0.015 (4)	0.061 (5)	−0.010 (4)
F6'	0.069 (3)	0.110 (5)	0.092 (5)	0.035 (3)	0.002 (3)	−0.022 (4)

### Geometric parameters (Å, °)

**Table:**

C26—C27	1.387 (2)	C19—H19	0.9300
C26—C25	1.391 (2)	C10—H10	0.9300
C26—C29	1.474 (2)	C1—C2	1.373 (3)
C22—C21	1.188 (2)	C1—H1	0.9300
C22—C23	1.439 (2)	C16—C17	1.364 (3)
C23—C24	1.385 (2)	C16—H16	0.9300
C23—C28	1.393 (2)	C17—C18	1.364 (3)
C12—C11	1.387 (2)	C17—H17	0.9300
C12—C13	1.394 (2)	C4—C3	1.364 (4)
C12—C30	1.474 (2)	C4—C5	1.385 (3)
C7—C8	1.192 (2)	C4—H4	0.9300
C7—C6	1.430 (2)	C18—H18	0.9300
C20—C19	1.380 (2)	C2—C3	1.350 (4)
C20—C15	1.388 (2)	C2—H2	0.9300
C20—C21	1.432 (2)	C5—H5	0.9300
C11—C10	1.370 (2)	C3—H3	0.9300
C11—H11	0.9300	C29—C30	1.347 (2)
C9—C14	1.380 (2)	C29—C33	1.490 (2)
C9—C10	1.390 (2)	C30—C31	1.498 (2)
C9—C8	1.435 (2)	C31—F2'	1.266 (17)
C27—C28	1.372 (2)	C31—F1'	1.350 (15)
C27—H27	0.9300	C31—F2	1.351 (7)
C28—H28	0.9300	C31—F1	1.380 (8)
C24—C25	1.376 (2)	C31—C32	1.522 (3)
C24—H24	0.9300	C33—F5'	1.251 (6)
C25—H25	0.9300	C33—F5	1.273 (4)
C6—C1	1.370 (3)	C33—F6	1.435 (5)
C6—C5	1.374 (3)	C33—C32	1.503 (3)
C14—C13	1.377 (2)	C33—F6'	1.546 (8)
C14—H14	0.9300	C32—F4'	1.151 (7)
C13—H13	0.9300	C32—F4	1.297 (4)
C15—C16	1.371 (3)	C32—F3	1.421 (4)
C15—H15	0.9300	C32—F3'	1.563 (11)
C19—C18	1.381 (3)		
			
C27—C26—C25	118.64 (15)	C3—C4—C5	120.3 (3)
C27—C26—C29	118.91 (14)	C3—C4—H4	119.9
C25—C26—C29	122.44 (15)	C5—C4—H4	119.9
C21—C22—C23	179.6 (2)	C17—C18—C19	120.2 (2)
C24—C23—C28	118.35 (16)	C17—C18—H18	119.9
C24—C23—C22	121.37 (15)	C19—C18—H18	119.9
C28—C23—C22	120.28 (16)	C3—C2—C1	120.4 (3)
C11—C12—C13	117.76 (15)	C3—C2—H2	119.8
C11—C12—C30	120.50 (14)	C1—C2—H2	119.8
C13—C12—C30	121.67 (14)	C6—C5—C4	119.8 (2)
C8—C7—C6	179.4 (2)	C6—C5—H5	120.1
C19—C20—C15	118.71 (17)	C4—C5—H5	120.1
C19—C20—C21	121.46 (16)	C2—C3—C4	119.9 (2)
C15—C20—C21	119.82 (16)	C2—C3—H3	120.0
C10—C11—C12	121.15 (15)	C4—C3—H3	120.0
C10—C11—H11	119.4	C30—C29—C26	128.39 (14)
C12—C11—H11	119.4	C30—C29—C33	111.11 (15)
C14—C9—C10	118.09 (16)	C26—C29—C33	120.24 (14)
C14—C9—C8	122.16 (16)	C29—C30—C12	128.56 (15)
C10—C9—C8	119.71 (16)	C29—C30—C31	110.29 (14)
C22—C21—C20	177.5 (2)	C12—C30—C31	121.10 (14)
C28—C27—C26	121.08 (15)	F2'—C31—F1'	109.8 (12)
C28—C27—H27	119.5	F2'—C31—F2	93.8 (10)
C26—C27—H27	119.5	F1'—C31—F1	119.5 (9)
C27—C28—C23	120.46 (16)	F2—C31—F1	103.8 (5)
C27—C28—H28	119.8	F2'—C31—C30	112.8 (9)
C23—C28—H28	119.8	F1'—C31—C30	108.2 (7)
C7—C8—C9	176.0 (2)	F2—C31—C30	115.1 (3)
C25—C24—C23	121.32 (15)	F1—C31—C30	112.2 (4)
C25—C24—H24	119.3	F2'—C31—C32	116.8 (10)
C23—C24—H24	119.3	F1'—C31—C32	103.1 (7)
C24—C25—C26	120.14 (16)	F2—C31—C32	113.1 (3)
C24—C25—H25	119.9	F1—C31—C32	107.1 (4)
C26—C25—H25	119.9	C30—C31—C32	105.42 (14)
C1—C6—C5	118.96 (19)	F5'—C33—F5	68.6 (5)
C1—C6—C7	120.71 (18)	F5—C33—F6	105.1 (2)
C5—C6—C7	120.3 (2)	F5'—C33—C29	117.4 (3)
C13—C14—C9	121.02 (16)	F5—C33—C29	118.5 (3)
C13—C14—H14	119.5	F6—C33—C29	108.1 (2)
C9—C14—H14	119.5	F5'—C33—C32	127.6 (5)
C14—C13—C12	120.90 (16)	F5—C33—C32	114.8 (3)
C14—C13—H13	119.5	F6—C33—C32	103.1 (3)
C12—C13—H13	119.5	C29—C33—C32	106.09 (15)
C16—C15—C20	120.58 (19)	F5'—C33—F6'	98.8 (4)
C16—C15—H15	119.7	F6—C33—F6'	135.2 (4)
C20—C15—H15	119.7	C29—C33—F6'	102.4 (4)
C20—C19—C18	120.1 (2)	C32—C33—F6'	98.9 (4)
C20—C19—H19	120.0	F4'—C32—F4	60.9 (10)
C18—C19—H19	120.0	F4—C32—F3	104.6 (3)
C11—C10—C9	121.03 (16)	F4'—C32—C33	122.9 (4)
C11—C10—H10	119.5	F4—C32—C33	119.7 (3)
C9—C10—H10	119.5	F3—C32—C33	105.1 (3)
C6—C1—C2	120.6 (2)	F4'—C32—C31	126.0 (6)
C6—C1—H1	119.7	F4—C32—C31	115.8 (2)
C2—C1—H1	119.7	F3—C32—C31	104.8 (2)
C17—C16—C15	120.0 (2)	C33—C32—C31	105.32 (16)
C17—C16—H16	120.0	F4'—C32—F3'	101.8 (7)
C15—C16—H16	120.0	F3—C32—F3'	145.5 (6)
C16—C17—C18	120.3 (2)	C33—C32—F3'	91.0 (6)
C16—C17—H17	119.8	C31—C32—F3'	99.8 (5)
C18—C17—H17	119.8		
			
C13—C12—C11—C10	−0.4 (3)	C26—C29—C33—F5'	30.1 (8)
C30—C12—C11—C10	176.79 (17)	C30—C29—C33—F5	136.2 (4)
C25—C26—C27—C28	−0.4 (2)	C26—C29—C33—F5	−49.2 (4)
C29—C26—C27—C28	178.34 (15)	C30—C29—C33—F6	−104.6 (3)
C26—C27—C28—C23	0.8 (3)	C26—C29—C33—F6	70.0 (3)
C24—C23—C28—C27	−0.4 (3)	C30—C29—C33—C32	5.4 (2)
C22—C23—C28—C27	−179.93 (16)	C26—C29—C33—C32	−179.99 (16)
C28—C23—C24—C25	−0.6 (3)	C30—C29—C33—F6'	108.6 (5)
C22—C23—C24—C25	179.00 (16)	C26—C29—C33—F6'	−76.8 (5)
C23—C24—C25—C26	1.0 (3)	F5'—C33—C32—F4'	−71 (2)
C27—C26—C25—C24	−0.5 (2)	F5—C33—C32—F4'	10.4 (18)
C29—C26—C25—C24	−179.20 (15)	F6—C33—C32—F4'	−103.3 (17)
C10—C9—C14—C13	−1.8 (3)	C29—C33—C32—F4'	143.2 (15)
C8—C9—C14—C13	176.01 (19)	F6'—C33—C32—F4'	37.4 (19)
C9—C14—C13—C12	−0.1 (3)	F5'—C33—C32—F4	1.7 (11)
C11—C12—C13—C14	1.2 (3)	F5—C33—C32—F4	83.1 (6)
C30—C12—C13—C14	−176.01 (17)	F6—C33—C32—F4	−30.6 (5)
C19—C20—C15—C16	−0.8 (3)	C29—C33—C32—F4	−144.1 (3)
C21—C20—C15—C16	177.85 (18)	F6'—C33—C32—F4	110.1 (7)
C15—C20—C19—C18	1.4 (3)	F5'—C33—C32—F3	−115.4 (10)
C21—C20—C19—C18	−177.18 (19)	F5—C33—C32—F3	−34.0 (5)
C12—C11—C10—C9	−1.5 (3)	F6—C33—C32—F3	−147.7 (4)
C14—C9—C10—C11	2.5 (3)	C29—C33—C32—F3	98.8 (3)
C8—C9—C10—C11	−175.31 (19)	F6'—C33—C32—F3	−6.9 (6)
C5—C6—C1—C2	0.7 (3)	F5'—C33—C32—C31	134.3 (8)
C7—C6—C1—C2	−179.4 (2)	F5—C33—C32—C31	−144.4 (4)
C20—C15—C16—C17	−0.5 (3)	F6—C33—C32—C31	102.0 (3)
C15—C16—C17—C18	1.1 (4)	C29—C33—C32—C31	−11.5 (2)
C16—C17—C18—C19	−0.5 (4)	F6'—C33—C32—C31	−117.3 (5)
C20—C19—C18—C17	−0.8 (3)	F5'—C33—C32—F3'	33.8 (13)
C6—C1—C2—C3	0.6 (4)	F5—C33—C32—F3'	115.2 (9)
C1—C6—C5—C4	−1.2 (4)	F6—C33—C32—F3'	1.5 (8)
C7—C6—C5—C4	178.9 (2)	C29—C33—C32—F3'	−112.0 (6)
C3—C4—C5—C6	0.4 (5)	F6'—C33—C32—F3'	142.3 (10)
C1—C2—C3—C4	−1.3 (4)	F2'—C31—C32—F4'	93.4 (19)
C5—C4—C3—C2	0.8 (5)	F1'—C31—C32—F4'	−27 (2)
C27—C26—C29—C30	52.6 (2)	F2—C31—C32—F4'	−13.8 (15)
C25—C26—C29—C30	−128.69 (18)	F1—C31—C32—F4'	99.9 (15)
C27—C26—C29—C33	−120.95 (18)	C30—C31—C32—F4'	−140.4 (15)
C25—C26—C29—C33	57.7 (2)	F2'—C31—C32—F4	21.9 (11)
C26—C29—C30—C12	11.8 (3)	F1'—C31—C32—F4	−98.6 (10)
C33—C29—C30—C12	−174.18 (16)	F2—C31—C32—F4	−85.4 (5)
C26—C29—C30—C31	−170.71 (15)	F1—C31—C32—F4	28.3 (6)
C33—C29—C30—C31	3.3 (2)	C30—C31—C32—F4	148.0 (4)
C11—C12—C30—C29	28.9 (3)	F2'—C31—C32—F3	136.5 (11)
C13—C12—C30—C29	−153.99 (18)	F1'—C31—C32—F3	16.0 (10)
C11—C12—C30—C31	−148.36 (17)	F2—C31—C32—F3	29.3 (4)
C13—C12—C30—C31	28.7 (2)	F1—C31—C32—F3	143.0 (5)
C29—C30—C31—F2'	118.0 (11)	C30—C31—C32—F3	−97.3 (3)
C12—C30—C31—F2'	−64.3 (11)	F2'—C31—C32—C33	−112.9 (10)
C29—C30—C31—F1'	−120.4 (8)	F1'—C31—C32—C33	126.7 (8)
C12—C30—C31—F1'	57.4 (8)	F2—C31—C32—C33	139.9 (3)
C29—C30—C31—F2	−136.0 (3)	F1—C31—C32—C33	−106.4 (4)
C12—C30—C31—F2	41.8 (3)	C30—C31—C32—C33	13.3 (2)
C29—C30—C31—F1	105.7 (5)	F2'—C31—C32—F3'	−19.1 (13)
C12—C30—C31—F1	−76.6 (5)	F1'—C31—C32—F3'	−139.6 (13)
C29—C30—C31—C32	−10.6 (2)	F2—C31—C32—F3'	−126.4 (8)
C12—C30—C31—C32	167.15 (16)	F1—C31—C32—F3'	−12.7 (8)
C30—C29—C33—F5'	−144.5 (8)	C30—C31—C32—F3'	107.0 (7)
